# Implementation and Impact of a Patient Blood Management Program in an Urban Community Hospital: An Eight-Year Study

**DOI:** 10.3390/healthcare13192462

**Published:** 2025-09-28

**Authors:** Robert Karpinos, Mark Friedman, Daniel Lombardi, Yahhua Li, Valdet Cobaj, Masooma Niazi, Phi Lai, Ding Wen Wu

**Affiliations:** 1 SBH Health, Bronx NY 10457, USAdlombardi@sbhny.org (D.L.);; 2 Department of Pathology, Transfusion Medicine Service, New York University Long Island School of Medicine, Mineola, NY 11501, USA; mark.friedman@nyulangone.org; 3 Department of Pathology and Laboratory Medicine, Tufts Medical Center, Boston, MA 02111, USA; yanhua.li@tuftsmedicine.org; 4 Department of Pathology, New York University Grossman School of Medicine, 550 1st Ave, New York, NY 10016, USA

**Keywords:** patient blood management, red blood cell transfusion, hemoglobin, cost savings, retrospective analysis

## Abstract

**Purpose**: This study evaluates the efficacy of a patient blood management (PBM) initiative in reducing unnecessary red blood cell (RBC) transfusions at a New York City community teaching hospital over eight years (2013–2020). **Methods**: A retrospective analysis of RBC transfusion data was performed, covering the period from 2013 to 2020. **Findings**: Post-PBM implementation, notable advancements were recorded annually. Mean pretransfusion hemoglobin (Hgb) levels decreased from 7.26 g/dL in 2013 to 6.68 g/dL in 2020. Annual RBC transfusion units decreased, with units transfused at Hgb ≥ 7 g/dL falling from 1210 (58.7%) in 2013 to 377 (20.0%) in 2020, a drop of 39%. Two-unit RBC orders at Hgb ≥ 7 g/dL declined from 65 in 2013 to 10 in 2020. Estimated cost savings from 2014 to 2020 totaled US Dollar (USD) 2.2 million. **Conclusions**: The PBM program significantly curtailed unnecessary RBC transfusions and optimized transfusion practices, demonstrating that resource-light, evidence-based strategies can yield substantial clinical and economic benefits.

## 1. Introduction

Adopting patient blood management (PBM) in a resource-constrained community hospital presents unique challenges. However, strategic interventions can effectively integrate PBM principles without requiring significant financial investment.

Located in a medically underserved area of New York City, a 350-bed urban community teaching hospital and acute care trauma center hosts diverse residency programs, including internal medicine, surgery, pediatrics, and emergency medicine. In 2013, this hospital launched a PBM program, fully operational by 2014, to enhance blood transfusion practices and patient outcomes through an evidence-based, multidisciplinary approach [1].

The PBM initiative aimed to minimize transfusion rates, improve patient care, and reduce costs by fostering collaboration among physicians, nurses, laboratory staff, and administrators. Key strategies included the following:

Educational outreach: Annual training modules were provided to clinicians, focusing on evidence-based blood management and updated transfusion guidelines [2]. Prophylactic transfusions are not recommended when hemoglobin (Hgb) levels are ≥7 g/dL, except in patients with coronary artery disease (CAD), for whom the threshold is raised to 8 g/dL. However, clinical symptoms of anemia take precedence over fixed Hgb thresholds in transfusion decisions.

Laboratory optimization: Transition to low-volume sample testing to reduce iatrogenic anemia and also decrease unnecessary blood draws.

Protocol adjustments: The critical Hgb reporting threshold was lowered from 7 g/dL to 6 g/dL, ensuring transfusions were driven by clinical need.

Transfusion ordering modifications: The default RBC orders were changed from 2 units to 1unit [3], curbing overuse.

Multidisciplinary collaboration: Implementation was through existing interdepartmental cooperation and teaching.

These measures, implemented without dedicated funding, leveraged existing resources and interdisciplinary commitment to achieve sustainable improvements in transfusion practices.

## 2. Methods

A retrospective review of RBC transfusions from 2013 to 2020 was conducted to assess the PBM program’s impact. The study received exempt status from the SBH Health Institutional Review Board.

### 2.1. Hypothesis

An evidence-based PBM program would significantly reduce unnecessary RBC transfusions.

### 2.2. Objective

To evaluate the PBM program’s effect on RBC transfusion practices and associated costs.

### 2.3. Data Inclusion and Exclusion Criteria

This study analyzed inpatient RBC transfusion data from 1 January 2013 to 31 December 2020, sourced from the hospital’s transfusion database. All inpatient RBC transfusion events were included, regardless of patient demographics (age, gender, race) or condition, except for the following:

- Emergency transfusions with uncrossmatched RBC for trauma or severe bleeding.
- Massive transfusion protocol events.
- Operation room (OR) transfusions.
- Outpatient transfusions.
- Transfusions involving plasma, platelets, or cryoprecipitate.

### 2.4. Study Outcomes

The following metrics were compared annually (2014–2020) against 2013 baseline data:

1. Mean and median pretransfusion Hgb levels triggering RBC orders.
2. Total annual RBC transfusion units.
3. Number of RBC units transfused at Hgb ≥ 7 g/dL (# Hb la7).
4. Percentage of RBC units transfused at Hgb ≥ 7 g/dL (% Hb la7).
5. Number of two-unit RBC orders.
6. Number and percentage of two-unit RBC orders at Hgb ≥ 7 g/dL.
7. Overall RBC transfusion rate (units per 1000 patient days, calculated as RBC transfusions ÷ (total discharges × average length of stay [LOS]).
8. Inpatient LOS (mean and median).
9. Cost savings from reduced unnecessary transfusions, estimated as
  - Potential reduction in RBC units = (% Hb la7 in 2013 − % Hb la7 in year of interest) × total RBC units in year of interest.
  - Cost savings = Potential reduction in RBC units × 760 USD per unit, reflecting the total transfusion-related cost per RBC unit, not merely the acquisition cost [4,5].

### 2.5. Statistical Analysis

Data were reported as mean ± standard deviation, with medians provided where relevant. To assess temporal trends across the study period, linear regression analyses were performed with year treated as a continuous variable. These analyses were complemented by non-parametric Mann–Kendall trend tests to further validate trend detection. Regression coefficients (β) with 95% confidence intervals were calculated to quantify annual changes in each outcome measure. Statistical analyses were performed using R software (version 4.3.1). A *p*-value < 0.05 indicated statistical significance.

### 2.6. Cost Sensitivity Analysis

Published cost-per-unit estimates (USD $522–1183 per unit in 2010) [4] were adjusted to 2013–2020 dollars to reflect price changes over the study period. Adjustments used (1) the Consumer Price Index for All Urban Consumers (CPI-U, all items) and (2) the CPI medical care component. For each year (*y*), the 2010 cost was multiplied by (CPI _y/CPI_2010) to obtain year-specific per-unit costs, which were then multiplied by units avoided to calculate annual savings. Results are reported for both CPIs as sensitivity bounds. The CPI series and monthly index values (July of each year) were obtained from the Bureau of Labor Statistics/FRED data tables [6,7].

## 3. Results

**Figure 1:**
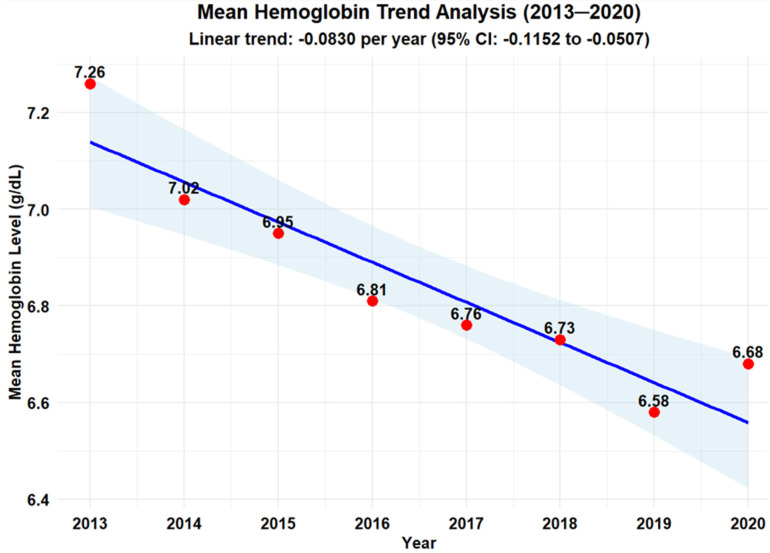
Mean hemoglobin level for RBC transfusion orders, 2013–2020. Note: Median Hgb for RBC transfusion orders revealed the same downward trend (Supplementary Data Figure S1).

The PBM program, fully implemented in 2014, drove significant improvements in RBC transfusion practices:

Pretransfusion Hgb Levels: The mean hemoglobin (Hgb) level decreased from 7.26 g/dL in 2013 to 6.58 g/dL in 2019 (*p* < 0.001), demonstrating a consistent year-over-year decline. Although there was a slight rebound to 6.68 g/dL in 2020 possibly due to the COVID-19 pandemic, the level remained significantly lower compared to in 2013, the year PBM implementation began. Our trend analysis revealed statistically significant annual decreases in mean RBC units transfused (β = −0.0830, 95% CI: −0.1152 to −0.0507, *p* = 0.0007) (Figure 1). The estimate is reported with 95% confidence intervals.

RBC Transfusion Volume: Annual RBC units decreased from 2061 in 2013 to 1350 in 2019, a 34.5% reduction. In 2020, RBC units increased to 1884 possibly due to the COVID-19 pandemic but remained 8.6% below the 2013 baseline of 2061 units (Table 1).

Transfusions at Hgb ≥ 7 g/dL: The percentage of red blood cell (RBC) units transfused at hemoglobin (Hgb) levels ≥ 7 g/dL decreased from 1210 (58.7%) in 2013 to 377 (20.0%) in 2020, reflecting a 38.7% reduction in unnecessary transfusions (Table 1). Despite an increase in total RBC transfusion volume in 2020, the proportion of unnecessary transfusions at Hgb ≥ 7 g/dL continued to decline.

**Figure 2:**
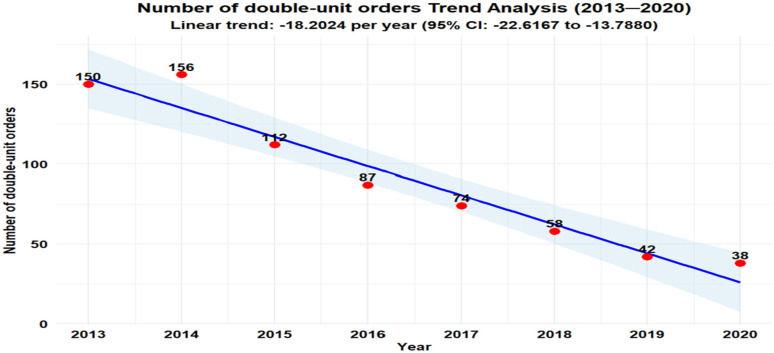
Number of double-unit orders.

**Figure 3:**
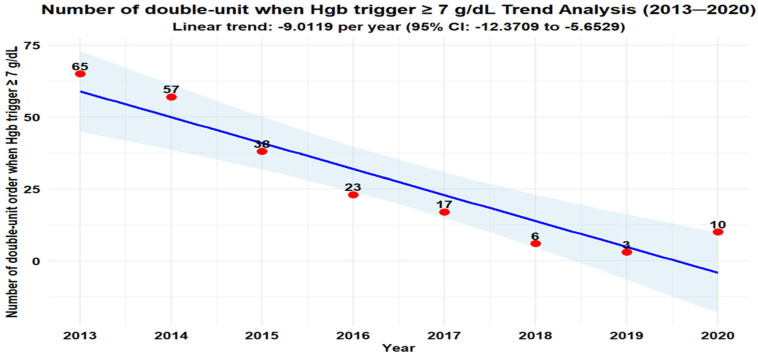
Number of double-unit orders when Hgb trigger ≥ 7 g/dL.

- Two-Unit Orders: Total two-unit RBC orders decreased from 150 in 2013 to 38 in 2020 (Figure 2). At Hgb ≥ 7 g/dL, two-unit orders are mostly unnecessary, dropping from 65 (3.4% of the two-unit transfusions) to 10 (0.7% of the two-unit transfusions) (Figure 3).

Our analysis revealed statistically significant annual decreases in the number of double-unit orders (β = −18.2024, 95% CI: −22.6167 to −13.7880, *p* = 0.0001) (Figure 2), and also the number of double-unit orders when the hemoglobin trigger was ≥7 g/dL (β = −9.0119, 95% CI: −12.3709 to −5.6529, *p* = 0.0006) (Figure 3). All estimates are reported with 95% confidence intervals.

**Figure 4:**
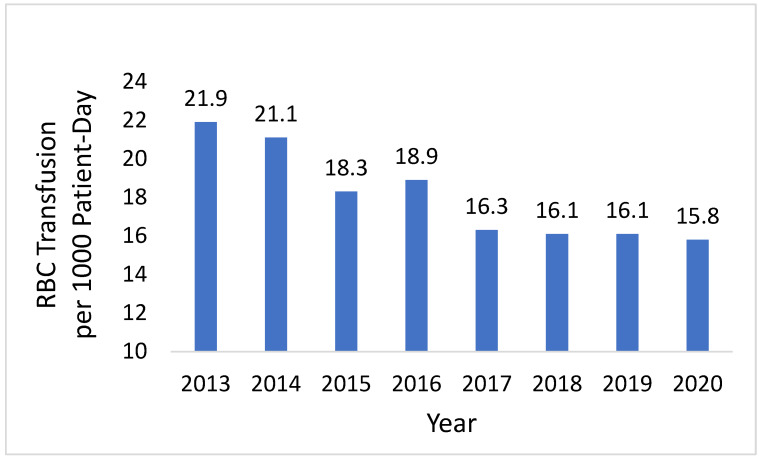
Annual inpatient overall RBC transfusion rate.

- Transfusion Rate: The annual inpatient overall RBC transfusion rate per 1000 patient days declined from 21.9 in 2013 to 15.8 in 2020 (Figure 4).

Overall rate of RBC transfusions = units of inpatient RBC transfusion without exclusions per 1000 inpatient days.

Length of Stay (LOS): Mean LOS remained stable (5.1 days in 2013 vs. 5.4 in 2020), with no change in median LOS (3.0 days), indicating no patient harm from restrictive practices (Table 2).

Estimated Cost Savings: The reduction in unnecessary RBC transfusions at hemoglobin levels ≥ 7 g/dL resulted in estimated savings of approximately 2844 units from 2014 to 2020, corresponding to ~USD 2.2 million in cost savings (Table 1), based on an estimated cost of ~USD 760 per unit [4].

Sensitivity Analysis Results Summary: Using the 2010 range in the literature (USD 522–1183/unit) and adjusting each year (2013–2020) for inflation,

○ All-item CPI adjustment → cumulative avoided transfusion cost over 2013–2020 ranges from USD 1.70 million (low bound) to USD 3.86 million (high bound).
○ Medical-care CPI adjustment → cumulative avoided cost over 2013–2020 ranges from USD 1.86 million (low bound) to USD 4.21 million (high bound).

The medical-care CPI produces larger adjusted costs (as expected) because medical prices rose faster than the all-item CPI over this interval.

## 4. Discussion

Overtransfusion poses risks including transfusion reactions, increased morbidity, higher costs, and strain on blood supplies [8,9]. Patient blood management (PBM) addresses these risks through evidence-based, patient-centered transfusion practices [1,8,9,10]. In this resource-limited community hospital, a PBM program without dedicated funding achieved a 34% reduction in RBC transfusions, aligning with global PBM goals.

Success was driven by low-cost strategies—clinician education, restrictive thresholds, low-volume testing, and EMR modifications [11]. Prioritizing symptomatic anemia over fixed hemoglobin (Hgb) triggers and lowering the Hgb threshold from 7 g/dL to 6 g/dL reduced unnecessary transfusions by 38.7% (from 58.7% to 20.0% of units at Hgb ≥ 7 g/dL). The “Why give 2 when 1 will do?” campaign [3,12] and CPOE alerts further decreased two-unit orders from 3.4% to 0.7%, consistent with Jenkins et al. [9].

These findings parallel outcomes from Warner et al., who reported a 33% transfusion reduction at a U.S. academic center [10], and Fischer et al., who demonstrated that education and behavioral change shift practice toward physiologic triggers [13]. Annual training reinforced clinician leadership and addressed barriers of limited PBM experience [14].

The World Health Organization’s 2025 policy brief on PBM underscores the global relevance of PBM findings [15]. Global evidence, including the Western Australia PBM Program involving >600,000 patients, shows reductions in mortality, infections, LOS, and substantial cost savings [14,15,16]. While smaller in scale, this study’s USD 2.1 million savings over eight years highlights the feasibility of PBM in community hospital settings without allocated budgetary support. The stable LOS (median, 3.0 days) suggests no patient harm, addressing concerns about restrictive transfusion risks, particularly for non-cardiac patients.

Comparative PBM studies reinforce these outcomes (Table 3).

### 4.1. Broader Implications and Future Directions

Our study shows that PBM can be effectively implemented in community hospitals without substantial funding by leveraging existing infrastructure, staff education, and EMR modifications. Sustained reductions in unnecessary transfusions reflect a culture shift consistent with multidisciplinary education and “Choosing Wisely” recommendations [2,3,12,13]. Building on this success, key strategies include (1) enhancing CPOE-CDS with smart best-practice alerts to further reduce inappropriate transfusions [18]; (2) integrating advanced analytics, including AI, to guide patient-specific transfusion thresholds; (3) fostering inter-institutional collaboration and knowledge sharing to extend training and best practices [14]; and (4) engaging patients in PBM to improve trust and compliance. These pragmatic, scalable approaches underscore PBM’s adaptability to resource-limited settings and provide a blueprint for broader adoption [11,15].

### 4.2. Challenges and Limitations

We attributed the increase in RBC transfusions in 2020 to the impact of the COVID-19 pandemic. This extraordinary period in healthcare led to the deprioritization of standard blood conservation practices in favor of urgent decision-making. Uncertainty regarding COVID-19 pathophysiology—particularly whether hypoxemia and thrombosis were pulmonary or hematologic—contributed to more liberal transfusion practices and likely increased utilization. Our PBM program for inpatients remained universal and inclusive, with consistent exclusions only for acute trauma, MTP, and OR cases, per ACS guidelines. However, we did not collect patient-level data identifying COVID-19–positive cases or their transfusion needs, limiting our ability to separate pandemic effects from other factors such as higher patient acuity, more frequent critical illness–related anemia, or demographic shifts. We now acknowledge this limitation and emphasize that future analyses should incorporate patient-level diagnostic data to better assess the pandemic’s influence on transfusion trends.

Observational design limits causal inference. Inclusion of neonates may have diluted reductions, while the exclusion of trauma, massive transfusion, and OR cases may introduce selection bias and limit the assessment of safety in high-risk groups. These choices allowed attention to be paid to patients most influenced by elective and medical decision-making but reduce comparability with overall hospital metrics. Reported volumes and savings may underestimate institutional impact, though including high-utilization groups could similarly dilute relative reductions. The CPOE-CDS system was also not fully optimized; prior work (e.g., that of Goodnough and Hollenhorst) demonstrated a 42% reduction in RBC use with best-practice alerts [17], suggesting further gains are possible.

Potential confounders must also be considered, including shifts in patient demographics, clinical complexity with broader safety endpoints (e.g., morbidity, case mix, readmission, cardiac events, ICU admission, mortality), and institutional factors such as evolving guidelines, staffing, and concurrent quality initiatives that may have influenced transfusion practices independent of PBM. Nonetheless, the sustained decline in unnecessary transfusions (Hgb ≥ 7 g/dL) over seven years suggests a robust PBM-driven culture change, even though the retrospective design cannot fully isolate the program’s impact from these potential confounders.

The program’s success also raises questions of scalability, as community hospitals with less robust EHRs or lower staff retention may face challenges, underscoring the need for external validation. While reductions in transfusions and costs are meaningful, they did not capture patient-centered outcomes (PROs) such as quality of life, recovery, or satisfaction. Moreover, without broader safety endpoints mentioned above, the overall impact on clinical safety remains uncertain. Although stable LOS and no documented transfusion-related harm suggest safety, future PBM studies should incorporate both clinical outcomes and PROs to fully assess effectiveness and patient-centered value.

Sustaining clinician engagement also remains challenging—Hofmann et al. identified limited PBM familiarity as a barrier [14]. High staff turnover and cultural resistance required continuous onboarding, peer-led discussions, and data-driven feedback. The decision to lower the Hgb critical threshold to 6 g/dL was evidence-based; although patients with comorbidities may require higher levels, outcomes showed no increased LOS or adverse events, supporting safety while warranting further study in high-risk groups. Together, these strategies fostered a sustained cultural shift in transfusion practice [11,14].

### 4.3. Cost Estimate

Our cost-saving calculations utilize a per-unit RBC cost of USD 760, drawn close from the mean of the activity-based cost range reported by Shander et al. [4]. Abraham and Sun [5] reported a cost of approximately USD 1225 for two RBC units. These adjustments provide a more defensible, evidence-based foundation for economic conclusions. The cost estimate of USD 760 per unit incorporates comprehensive direct and indirect costs, including acquisition, processing, labor, overhead, and administration. It predates more recent systemic changes and cost escalations.

Cost savings were inflation-adjusted by converting 2010 research estimates into year-specific dollars using CPI-U and medical care CPI indices. This standard approach has limits, as neither index is transfusion-specific. July values were applied, with annual averages yielding similar results. Because institution-specific costs were unavailable, savings reflect published per-unit estimates and should be interpreted as sensitivity bounds rather than exact hospital costs.

Our analysis indicates the point estimate (USD 2.2 million; ~USD 760 per unit) is robust under plausible inflation adjustments and conservative assumptions, falling in the lower-to-middle range of CPI-adjusted bounds. Savings could be substantially higher if broader medical care cost indices are used. The analysis also underscores uncertainty from index choice, suggesting future evaluations should use site-specific or updated activity-based costing data.

## 5. Conclusions

This eight-year retrospective study demonstrates the effectiveness of a patient blood management (PBM) program in reducing unnecessary red blood cell (RBC) transfusions at hemoglobin (Hgb) levels ≥ 7 g/dL by 38.7%, yielding estimated cost savings of approximately USD 2.1 million in a resource-constrained community hospital without dedicated funding. No evidence of patient harm was observed. By leveraging clinician education, evidence-based restrictive transfusion guidelines, and electronic medical record tools, this program provides a scalable model for optimizing transfusion practices in similar settings.

## Figures and Tables

**Table 1:** RBC units transfused and potential cost savings.

Year	2013	2014	2015	2016	2017	2018	2019	2020	Total
(1) Number of transfused RBC units	2061	1762 *	1460 *	1514 *	1251 *	1286 *	1350 *	1884	N/A
(2) Number of RBC units transfused when Hgb trigger ≥ 7 g/dL	1210	830 *	572 *	517 *	371 *	357 *	310 *	377 *	N/A
(3) % of RBC units transfused when Hgb trigger ≥ 7 g/dL = (2) ÷ (1)	58.7	47.1	39.2	34.1	30.0	27.8	23.0	20.0	N/A
(4) Potential reduction in RBC units = ((3) of year 2013 − (3) of this year) × (1) of the year) **	N/A	204	285	372	375	398	482	729	2844
(5) Potential cost saving (USD) = (4) × USD 760 per unit	N/A	155 K	217 K	283 K	285 K	302 K	366 K	554 K	USD 2.2 million

* *p*-value < 0.001 compared to the number in year 2013. ** Row (4) calculation example: potential reduction in RBC units in 2020 = (58.7% − 20.0%) × 1884 = 729 units. Cost savings calculated as ~USD $760 per unit [4].

**Table 2:** Hospital inpatients’ length of stay (LOS).

LOS (Days)	2013	2014	2015	2016	2017	2018	2019	2020
Mean (Days)	5.1	4.9	5.5	5.5	5.2	5.3	5.5	5.4
Median (Days)	3.0	3.0	3.0	3.0	3.0	3.0	3.0	3.0

**Table 3:** Comparative PBM studies.

Author/Study	Focus/Topic	Methodology	Key Findings	Limitations
Fischer et al. (2015) [13]	PBM education and behavioral change	Observational study (within 2 years)	Shift toward physiological transfusion triggers	Physician perception-based; limited outcome data
Froessler et al. (2016) [17]	Preoperative IV iron therapy	Randomized controlled trial (over 3+ years)	Reduced transfusions (61%), improved recovery, shorter stays, cost saving	Specific to surgical patients; not generalizable to all PBM contexts
Jenkins et al. (2017) [9]	Clinical decision support for transfusions	Quality improvement study	Significant reduction in double-unit orders	Limited to one institution; no long-term follow-up
Leahy et al. (2017) [16]	System-wide PBM in Australia, involving over 600,000 patients	A 6-year retrospective study across 4 tertiary-care hospitals	Reduced transfusions (41%), mortality, infections, LOS, and product costs	Large-scale system; may not reflect smaller hospital settings
Goodnough & Hollenhorst (2019) [18]	Smart alerts in CPOE for PBM	An 11-year study for the impact of PBM CDS	42% reduction in RBC use	Requires advanced EHR systems; scalability concerns
Warner et al. (2019) [10]	Single-unit default in electronic ordering	An 8-year observational study at a medical center	33% reduction in RBC transfusions	Focused on academic center; may not generalize to smaller hospitals
Wu et al. (2024) [11]	PBM in a community hospital, without allocation of budgetary support	A 7-year retrospective study	35.7% reduction in unnecessary RBC transfusions; cost savings; no harm to patients	Retrospective design; no patient-level COVID data; limited generalizability

## Data Availability

The original contributions presented in this study are included in the article/Supplementary Material. Further inquiries can be directed to the corresponding authors.

## Supplementary Materials

The following supporting information can be downloaded at https://www.mdpi.com/article/10.3390/healthcare13192462/s1: Figure S1: Median hemoglobin levels for RBC transfusion orders (*p* < 0.001 vs. year 2013).
