# Supplementary material for: Implementation and Impact of a Patient Blood Management Program in an Urban Community Hospital: An Eight-Year Study
Source: Healthcare (Basel). 2025 Sep 28;13(19):2462. doi: 10.3390/healthcare13192462 (PMC12524869; doi:10.3390/healthcare13192462)
Supplement: Supplementary file 1 [file healthcare-13-02462-s001.zip › healthcare-3776874-supplementary.pdf]

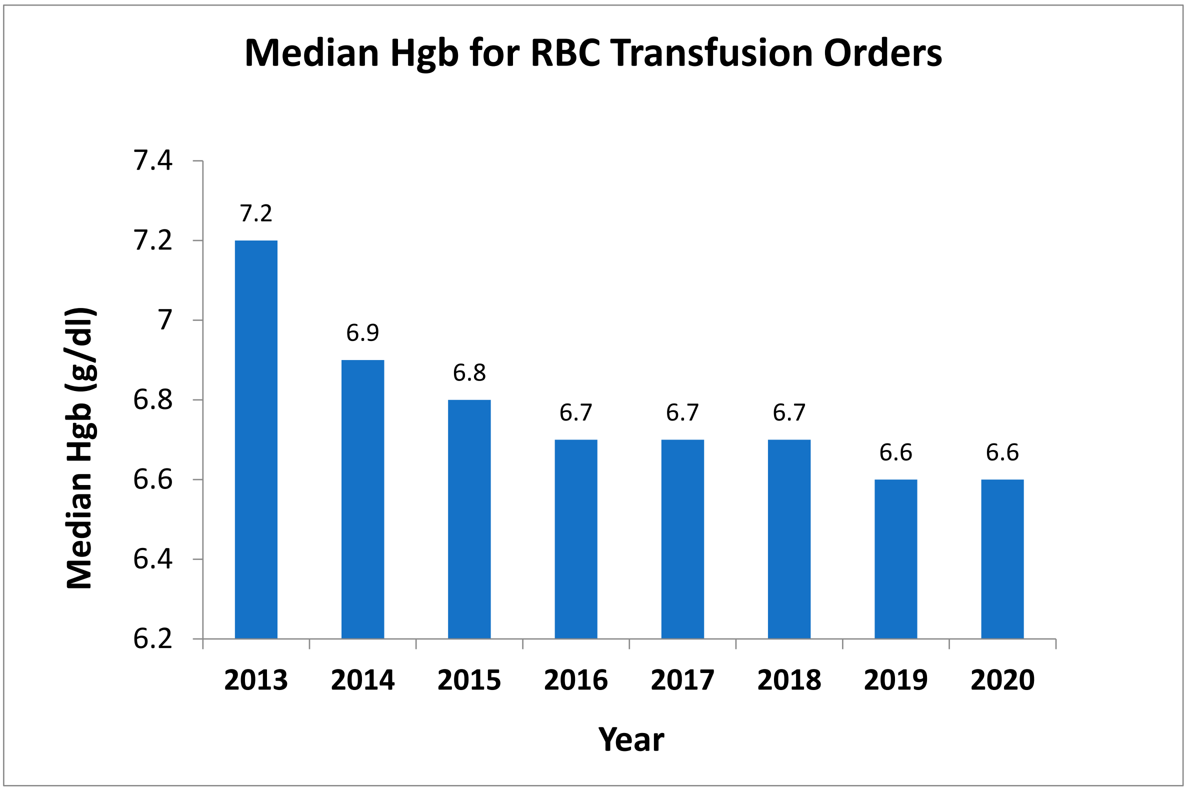

Figure S1. Median Hemoglobin Levels for RBC Transfusion Orders ($p <$ 0.001 vs. year 2013).

The p-value is < 0.001 for the value of each year from 2014 to 2020 compared to the value of 2013.
